# The Impact of COVID-19 Pandemic in Portuguese Cancer Patients: A Retrospective Study

**DOI:** 10.3390/ijerph18168552

**Published:** 2021-08-13

**Authors:** Aurea Lima, Hugo Sousa, Amanda Nobre, Ana Luisa Faria, Manuela Machado

**Affiliations:** 1 Centro Hospitalar de Entre o Douro e Vouga EPE, Medical Oncology Department, Unit of Santa Maria da Feira, Rua Dr. Cândido Pinho 5, 4520-211 Santa Maria da Feira, Portugal; amanda.carvalho@chedv.min-saude.pt (A.N.); ana.faria@chedv.min-saude.pt (A.L.F.); manuela.machado@chedv.min-saude.pt (M.M.); 2 Research Center (CI-IPOP)/RISE@CI-IPOP (Health Research Network), Porto Comprehensive Cancer Center (Porto.CCC), Molecular Oncology and Viral Pathology Group, Portuguese Oncology Institute of Porto (IPO Porto), Rua Dr. António Bernardino de Almeida, 4200-072 Porto, Portugal; hugo.sousa@ipoporto.min-saude.pt; 3 Cooperativa de Ensino Superior Politécnico e Universitário (CESPU), Department of Pharmaceutical Sciences, Institute of Research and Advanced Training in Health Sciences and Technologies, Rua Central de Gandra 1317, 4585-116 Gandra, Portugal; 4 Virology Service, Portuguese Institute of Oncology of Porto (IPO-Porto), Rua Dr. António Bernardino de Almeida, 4200-072 Porto, Portugal

**Keywords:** COVID-19, SARS-CoV-2, coronavirus, neoplasms, cancer, oncology

## Abstract

Literature reports that SARS-CoV-2 infection in cancer patients may be associated with higher severity and mortality, nevertheless the knowledge is limited. We aimed to describe patients’ demographic characteristics and COVID-19 disease outcomes in Portuguese cancer patients. We conducted a retrospective study in a cohort of cancer patients diagnosed with COVID-19. A total of 127 individuals were included: 46.5% males and 53.5% females, with a median age of 72 years. Clinicopathological characteristics were used in univariate and multivariable logistic regression analyses to estimate odds ratios for each variable with outcomes adjusting for potential confounders. Our cohort revealed that 84.3% of patients had more than one risk factor for severe disease rather than cancer. In total, 36.2% of patients were admitted to the Department of Internal Medicine, 14.2% developed severe disease, 1.6% required Intensive Care Unit, and mortality was observed in 11.8%. Severe COVID-19 disease was associated with unfit (ECOG PS > 2) patients (*p* = 0.009; OR = 6.39; 95% CI: 1.60–25.59), chronic kidney disease (*p* = 0.004; OR = 20.7; 95% CI: 2.64–162.8), immunosuppression (*p* < 0.001; OR = 10.3; 95% CI: 2.58–41.2), and presence of respiratory symptoms at diagnosis (*p* = 0.033; OR = 5.05; 95% CI: 1.14–22.4). Increased risk for mortality was associated with unfit patients (*p* = 0.036; OR = 4.22; 95% CI: 1.10–16.3), cardiac disease (*p* = 0.003; OR = 8.26; 95% CI: 2.03–33.6) and immunosuppression (*p* = 0.022; OR = 5.06; 95% CI: 1.27–20.18). Our results demonstrated that unfit and immunosuppressed patients, with chronic kidney disease and cardiac disease, have, respectively, an increased risk for severe disease and mortality related to COVID-19. Hence, this study provides important information on risk factors for severe COVID-19 disease and associated mortality in a Portuguese cancer population.

## 1. Introduction

A novel coronavirus known as severe acute respiratory syndrome coronavirus 2 (SARS-CoV-2) has widely spread across the world since its first reported case in late December 2019 in Wuhan, China [1]. The World Health Organization (WHO) promptly declared the coronavirus disease of 2019 (COVID-19) a public health emergency of international concern [2]. Since March 2020, this virus has affected more than 150M individuals causing more than 3.2M deaths in 220 countries and territories [2]. Nevertheless, the true level of SARS-CoV-2 transmission is probably underestimated since a substantial proportion of infected individuals are not identified either because they are asymptomatic or have only mild symptoms which directly impacts the number of diagnostic tests performed [3].

It has been described that approximately 15% of infected individuals develop severe disease, requiring hospitalization and oxygen support, while 5% develop critical disease with admission at Intensive Care Unit (ICU) [4]. Older age, smoking, and underlying noncommunicable diseases, such as diabetes, hypertension, cardiac disease, chronic lung disease, and cancer, have been reported as risk factors for severe disease and ultimately death [5,6,7,8]. Differences in COVID-19 severity and mortality between groups of people and countries are important proxy indicators of relative risk of death that guide policy decisions regarding scarce medical resource allocation during the ongoing COVID-19 pandemic.

With more than 18 million new cases per year globally, cancer affects a significant portion of the population [9]. Cancer patients are more susceptible to infections due to coexisting chronic diseases, overall poor health status, and systemic immunosuppressive states caused by both cancer and anticancer treatments [10]. Therefore, cancer patients infected by SARS-CoV-2 are expected to have more poor outcomes [11,12,13,14,15]. People with blood cancers may be at higher risk of prolonged infection and death from COVID-19 than people with solid tumors since these patients often have abnormal or depleted levels of immune cells affecting the ability to develop an immune response to viral infections [16]. Moreover, and also due to immunosuppression, some oncology treatments—such as chemotherapy, hematopoietic stem cell transplant, or cell therapy—are associated with an increased risk for SARS-CoV-2 infection and a more severe COVID-19 disease [17,18]. Nevertheless, and until now, the clinical characteristics of SARS-CoV-2-infected cancer patients, as well as the degree of infection severity and the impact of COVID-19 mortality in cancer patients, remains largely unknown. We aimed to describe the demographic characteristics and COVID-19 disease outcomes of cancer patients attended at Centro Hospitalar de Entre o Douro e Vouga (CHEDV) during the year 2020.

## 2. Materials and Methods

### 2.1. Design/Patient Population

This study retrospectively describes a cohort of cancer patients over 18 years old consecutively admitted to CHEDV during the year 2020 who tested positive for SARS-CoV-2. Cases were considered if had a positive real-time polymerase chain reaction (RT-PCR) test from a nasal or throat swab and, when available, a radiological or clinical diagnosis of COVID-19. From March 17 to 31 December of 2020 a total of 2755 CHEDV patients tested positive for SARS-CoV-2, of which 127 (4.7%) were cancer patients.

SARS-CoV-2 laboratory diagnosis was performed using Seegene^®^ Allplex 2019-nCoV Assay (Seegene Inc., Seoul, Korea). This assay targets four viral genes (E, N, and S/RdRp), and results were analyzed using Seegene Viewer software (Seegene Inc., Seoul, Korea).

### 2.2. Data Collection

Clinicopathological data (e.g., patient demographics, treatment details, COVID-19 disease course, and cancer features) were obtained from hospital individual clinical records and anonymized. Eastern Cooperative Oncology Group performance status (ECOG PS) grade was used as a patient performance scale [19]. Cancer type was defined according to the International Classification of Disease 10th Revision diagnostic codes (ICD-10), and tumor stage and grade were clustered according to the 8th edition of the American Joint Committee on Cancer (AJCC) Cancer Staging Manual [20]. Risk factors for severe COVID-19 disease, as well as COVID-19 severity category, were determined according to WHO guidelines [21]. A primary endpoint of all-cause mortality was defined to include deaths described as related to COVID-19 during admission, as well as deaths reported because of any other cause during admissions, such as due to cancer progression or treatment toxicity. Outcomes were measured on 30 April 2021. Patients were grouped using the two waves of incidence of coronavirus infection (March to August and September to December) for characterization.

### 2.3. Statistical Analysis

Data management and statistical analysis were performed using IBM^®^ SPSS^®^ Statistics version 26.0 (IBM Corp. in Armonk, NY, USA). Categorical variables were expressed using counts and percentages. Continuous data were presented as mean with standard deviation (±SD) or as median with range. The Chi-square test was used to assess the association between groups and different categorical variables considering a 5% or less statistically significant *p*-value. Age as a continuous variable was compared using a T-test. The threshold of ECOG PS chosen for defining unfitness was a value > 2. We assessed whether the patient died or eventually achieved discharge and observed the effect of all variables in the severity of the disease (comparing mild/moderate vs. severe disease) and mortality due to COVID-19. Logistic regression was used to perform univariate analysis, and the statistically significant variables were considered as candidates for the multivariable logistic model. Multivariable logistic regression was used to estimate the odds ratio (OR)s and 95% confidence intervals (CI)s adjusting for clinically relevant potential confounders (age > 60 years and gender).

Power of analysis was calculated using JAMOVI 1.8.1.0 software. The analysis revealed that a design with a sample size of *n* = 127 can detect effect sizes > 0.5 with a probability of 1.00 assuming a maximum type I error of 0.05.

### 2.4. Ethical Considerations

This work was approved by the Ethics Committee of CHEDV, according to the Helsinki Declaration of the World Medical Association.

## 3. Results

### 3.1. Patients’ Characteristics

**Figure 1:**
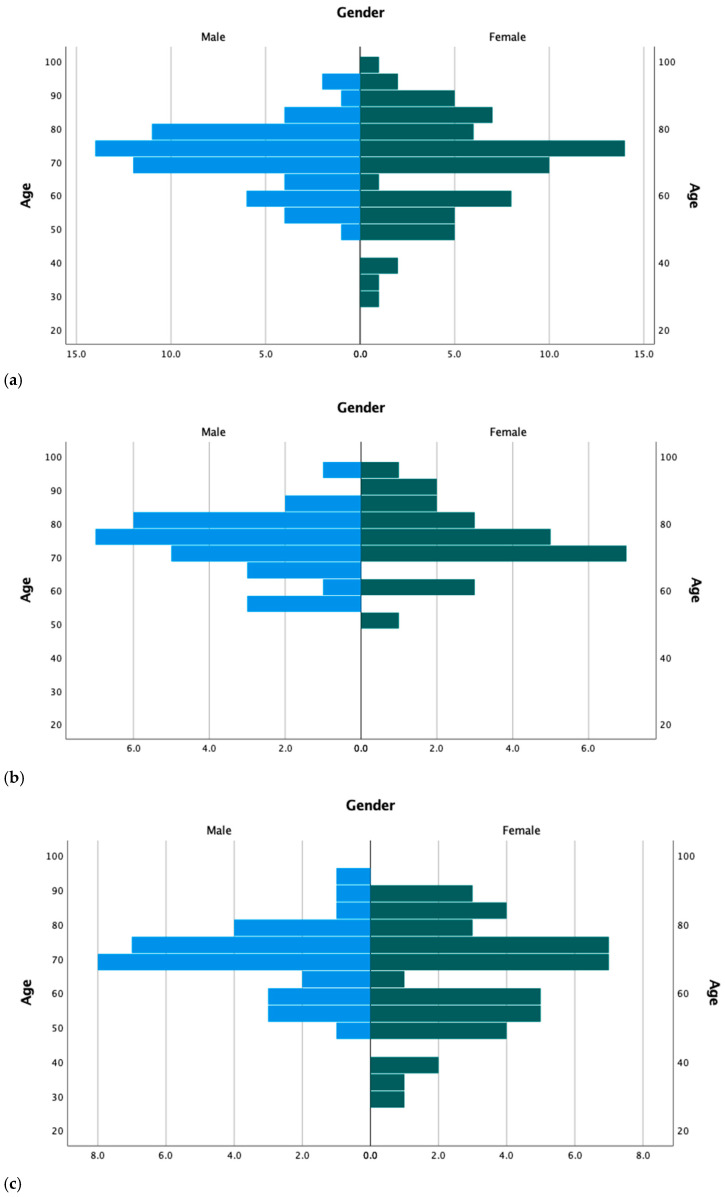
Distribution of patients by age groups and gender for the whole population (**a**), first (**b**), and second (**c**) pandemic waves.

Patients’ characteristics data are shown in Table 1. Our cohort included a total of 59 (46.5%) males and 68 (53.5%) females, with a median age of 72 years (range 29–100). Briefly, patients were mostly from the county of Santa Maria da Feira (*n* = 62; 48.8%), only 11 (8.7%) patients were elderly care residents, and almost 87% of patients presented other comorbidities beyond cancer. The cohort includes 53 (41.7%) patients from the first wave and 74 (58.3%) patients from the second wave of SARS-CoV-2 infection in Portugal. The median age at diagnosis of SARS-CoV-2 infection was statistically significantly different when comparing the first and second pandemic waves (median 74 years vs. 69 years; *p* = 0.002)—Figure 1.

Regarding the characteristics of the oncologic disease, patients presented a median ECOG PS of 1 (range 0–4) and the median time of oncological disease was 4.19 years (range 0.17–21.42); the most frequent tumors were digestive (27.6%), breast (24.4%) and urologic (17.3%), while hematological tumors represented 8.7% of the tumors. Of the patients with non-hematological tumors, 57.4% presented a tumor at an initial stage, 19.1% had locally advanced tumors, and 23.5% presented metastatic disease. Within the four weeks before testing positive for SARS-CoV-2 patients were under: palliative chemotherapy (*n* = 31; 25.8%), adjuvant chemotherapy (*n* = 32; 26.7%) and supportive care (*n* = 7; 5.8%).

### 3.2. COVID-19 Disease Characteristics

COVID-19 disease characteristics data are shown in Table 2. Considering WHO risk factors to severe SARS-CoV-2 infection, 20 (15.7%) patients had no other risk factor rather than being a cancer patient, 107 (84.2%) patients had more than 1 risk factor, and the maximum of risk factors was 6 (*n* = 1; 0.8%). SARS-CoV-2 infection was detected in the emergency environment (*n* = 66; 52.0%), screening for routine procedures (*n* = 42; 33.0%), and during hospitalization (*n* = 19; 15.0%). Infection was considered nosocomial in 22 (17.3%) patients. Of the 127 patients with SARS-CoV-2 infection, 41 (32.3%) showed no symptoms at the time of testing positive. Among the 86 symptomatic patients, symptoms related to the respiratory tract were the most observed (*n* = 62; 72.1%).

**Figure 2:**
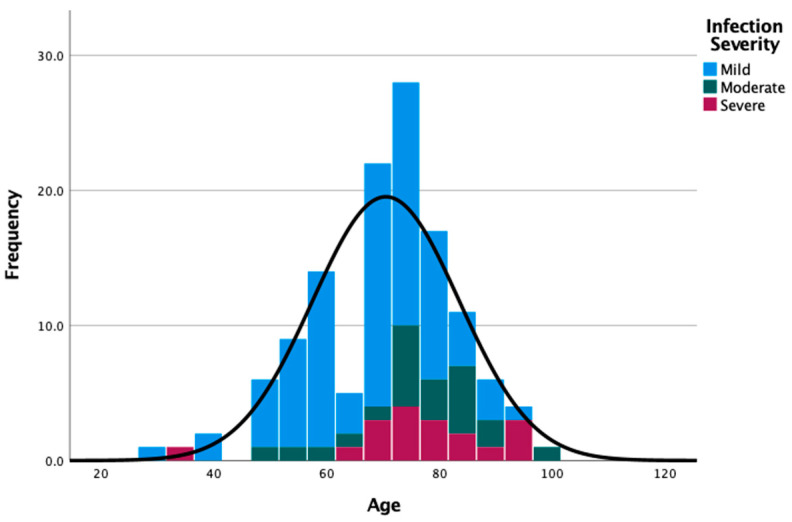
Distribution of patients by age groups and COVID-19 severity.

**Figure 3:**
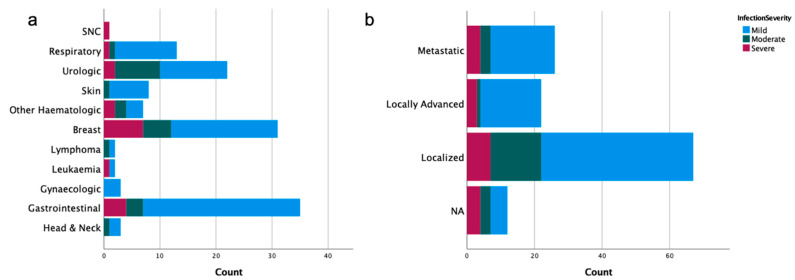
Oncological characteristics distributed by COVID-19 severity: (**a**) type of oncological tumor; and (**b**) stage of the disease.

COVID-19 severity was categorized as mild (*n* = 87, 68.5%), moderate (*n* = 22, 17.3%) and severe (*n* = 18, 14.2%). Patients’ age according to COVID-19 severity were as follows: median 70.0 (range 29–96) for mild, median 76.5 (range 49–100) for moderate, and median 76.0 (range 34–96) for severe cases. Forty-six (36.2%) patients were admitted to the Department of Internal Medicine, of which two (1.6%) patients required hospitalization in ICU. The median hospitalization duration was of 9.74 days (range 1.00–40.0). The distribution of patients according to age and COVID-19 severity is shown in Figure 2 and the oncological characteristics distributed according to COVID-19 severity are shown in Figure 3 (descriptive data can be found in Supplementary Materials Table S1).

**Figure 4:**
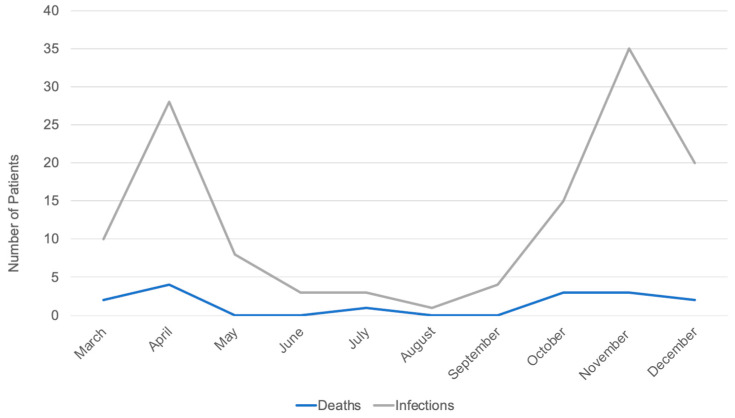
Evolution of SARS-CoV-2 infection and deaths due to COVID-19 disease.

**Figure 5:**
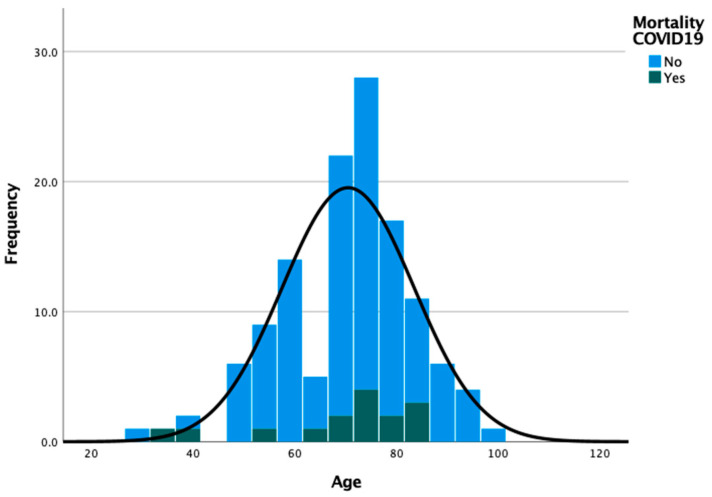
Distribution of patients by age groups and COVID-19 mortality.

**Figure 6:**
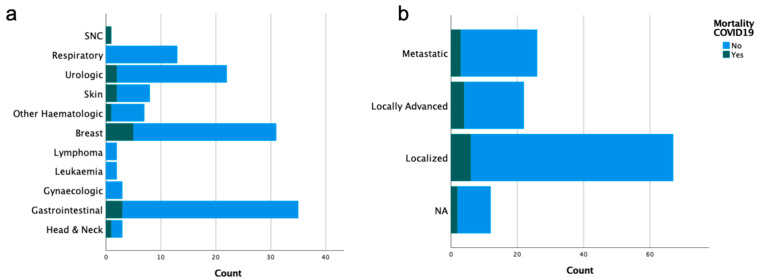
Oncological characteristics distributed by COVID-19 mortality: (**a**) type of oncological tumor; and (**b**) stage of the disease.

The evolution of SARS-CoV-2 infections and associated deaths is represented in Figure 4. Mortality due to COVID-19 disease was observed in 15 (11.8%) patients. The distribution of patients according to age groups and COVID-19 mortality is shown in Figure 5 and the oncological characteristics distributed by COVID-19 mortality are shown in Figure 6 (descriptive data can be found in Supplementary Materials Table S1).

### 3.3. COVID-19 Severe Disease in Cancer Patients

We performed a risk analysis to evaluate the impact of the different clinicopathological variables (patients and CODIV-19 disease related) on the development of severe COVID-19 disease (data not shown). The analysis revealed the following associations: elderly care patients (*p* < 0.001); chronic renal disease (*p* = 0.001); immunosuppression (*p* < 0.001); having ≥ 3 risk factors for severe disease (*p* = 0.014); having ≥ 4 risk factors for severe disease (*p* = 0.019); having ≥ 5 risk factors for severe disease (*p* = 0.025); ECOG PS > 2 (*p* = 0.001); symptomatic disease (*p* = 0.002); respiratory symptoms (*p* = 0.010); cough (*p* = 0.011); and dyspnea (*p* = 0.004). Borderline results were observed for: age > 60 years (*p* = 0.070); cardiac disease (*p* = 0.053); and conscience status changes (*p* = 0.089) variables.

The univariate analysis for the association of clinicopathological characteristics and severe COVID-19 are shown in Table 3. Severe COVID-19 disease was associated with elderly care patients (*p* < 0.001; OR = 10.2; 95% CI 2.70–38.5); chronic renal disease (*p* = 0.003; OR = 8.00; 95% CI 2.04–31.4); immunosuppression (*p* < 0.001; OR = 11.1; 95% CI 3.52–35.0); having ≥ 3 risk factors for severe disease (*p* = 0.018; OR = 3.47; 95% CI 1.24–9.72); ECOG PS > 2 (*p* = 0.002; OR = 5.41; 95% CI 1.88–15.6); respiratory symptoms (*p* = 0.013; OR = 4.45; 95% CI 1.38–14.4); cough (*p* = 0.014; OR = 3.74; 95% CI 1.30–10.70); and dyspnea (*p* = 0.007; OR = 4.19; 95% CI 1.48–11.8). Borderline results were observed for: age > 60 years (*p* = 0.084; OR = 6.17; 95% CI 0.78–48.4); cardiac disease (*p* = 0.061; OR = 2.91; 95% CI 0.95–8.86); and conscience status changes (*p* = 0.082; OR = 3.17; 95% CI 0.86–11.7) variables.

Multivariate analysis adjusted to gender and age > 60 years, analyzing the impact of the risk factors for severe disease and individual symptoms at diagnosis, confirmed that a severe COVID-19 disease was associated with unfit patients (*p* = 0.009; OR = 6.39; 95% CI: 1.60–25.59); chronic kidney disease (*p* = 0.004; OR = 20.7; 95% CI: 2.64–162.8); immunosuppression (*p* < 0.001; OR = 10.3; 95% CI: 2.58–41.2); and presence of respiratory symptoms at diagnosis (*p* = 0.033; OR = 5.05; 95% CI: 1.14–22.4). In our study, type of cancer, time of oncological disease, stage of cancer, and/or anticancer treatment before SARS-CoV-2 infection were not associated with COVID-19 severity.

### 3.4. COVID-19 Mortality in Cancer Patients

We performed a risk analysis to evaluate the impact of the different clinicopathological variables (patients and COVID-19 disease related) on the mortality due to COVID-19 disease (data not shown). The analysis revealed the following associations: cardiac disease (*p* = 0.001); immunosuppression (*p* = 0.002); having ≥4 risk factors for severe disease (*p* = 0.037); ECOG PS > 2 (*p* = 0.007); symptomatic disease (*p* = 0.003); fever (*p* = 0.041); conscience status changes (*p* < 0.001); and COVID-19 severe disease (*p* < 0.001) variables.

The univariate analysis for the association of clinicopathological characteristics and mortality due to COVID-19 are shown in Table 3. Mortality due to COVID-19 disease was associated with cardiac disease (*p* = 0.003; OR = 5.66; 95% CI 1.79–17.89); immunosuppression (*p* = 0.005; OR = 5.56; 95% CI 1.68–18.33); having ≥ 4 risk factors for severe disease (*p* = 0.033; OR = 3.48; 95% CI 1.10–10.99); ECOG PS > 2 (*p* = 0.011; OR = 4.28; 95% CI 1.39–13.23); fever (*p* = 0.049; OR = 3.04; 95% CI 1.01–9.18); conscience status changes (*p* < 0.001; OR = 15.46, 95% CI: 4.19–57.06); and COVID-19 severe disease (*p* < 0.001; OR = 11.6; 95% CI: 3.50–38.87) variables.

Multivariate analysis adjusted for gender and age > 60 years, analyzing the impact of the risk factors for severe disease, confirmed an increased risk for mortality associated with unfit patients (*p* = 0.036; OR = 4.22; 95% CI: 1.10–16.3); cardiac disease (*p* = 0.003; OR = 8.26; 95% CI: 2.03–33.6); and immunosuppression (*p* = 0.022; OR = 5.06; 95% CI: 1.27–20.18). Type of cancer, time of oncological disease, stage of cancer, and/or anticancer treatment before SARS-CoV-2 infection were not related to COVID-19 mortality.

## 4. Discussion

In the past months, SARS-CoV-2 infection has spread rapidly globally, affecting millions of people and with higher impact in individuals with significant comorbidities [18,22,23,24]. Cancer is among the top causes of death, and Europe is one of the continents with the highest incidence of cancer in the world [9]. Since the beginning of the SARS-CoV-2 pandemic, cancer has been recognized as an important risk factor for severe SARS-CoV-2 infection and COVID-19-associated mortality [6,8,10]. Compared with the general population, cancer patients are more vulnerable to infection [11,14]. A few studies have started to be presented showing data on the impact of the COVID-19 disease in cancer patient outcomes, especially in populations from China. A retrospective study from China has shown that patients with cancer and COVID-19 seemed to have a higher risk of COVID-19 complications, and worse outcomes [25]. In addition to the immunosuppressive states directly caused by cancer or cancer treatments, these patients are often older with overall poorer performance status, and with coexisting medical conditions. These are risk factors that could contribute to COVID-19 infection and lead to a potentially poorer prognosis and an increased risk of death in cancer patients.

Despite all the knowledge of COVID-19 in the general population, limited information has been reported about the outcome of cancer patients who are infected by SARS-CoV-2 [8,12]. Here, we intended to evaluate the outcome of a Portuguese cohort of cancer patients infected with SARS-CoV-2, by analyzing the potential risk factors for disease severity and mortality. Despite our study representing a midsize cohort, it will provide much-needed information on the risk factors of our population.

In our study, more than half of SARS-CoV-2 infections in cancer patients were detected in the emergency environment. Nevertheless, in a considerable group of patients, SARS-CoV-2 infection was detected in a screening context of asymptomatic patients prior to cancer treatments. These data reinforce the importance of screening cancer patients before being exposed to immunosuppressive treatments [26]. Furthermore, these data highlight the fact that the true level of SARS-CoV-2 infection is frequently underestimated. As expected, among the 86 symptomatic patients, symptoms related to the respiratory tract were the most observed [12]. Indeed, our results demonstrated that patients presenting respiratory symptoms at diagnosis had a 5-fold increased risk for severe disease. Severe disease was observed in 14.2% of the patients and only 1.6% required ICU. Literature supports this evidence since approximately 15% of infected patients are expected to develop severe disease, and that 5% will develop critical disease [15]. Mortality due to COVID-19 disease was observed in 15 (11.8%) patients. Although COVID-19 is reported to have a relatively low death rate of 2% to 3% in the general population [2], studies have shown that patients with cancer and COVID-19 have a nearly 3-fold increase in death rate than that of COVID-19 patients without cancer [11], which is in accordance to our results.

In our cohort, we observed that both genders were equally affected by SARS-CoV-2, with a median age of 72 years. Cancer is an aging disease and so, it is expected that cancer patients will be older than the population without cancer [27]. Nevertheless, statistically significant differences were observed regarding patients’ age in the first vs. second pandemic wave. Two characteristics were observed and deserve some reflection: (1) in the first wave patients were older and some of them were elderly care patients, which can explain the form of contamination; and (2) in the second wave patients were younger and the transmission form seems to be related to socialization. A recently published meta-analysis revealed that age over 65 years and male gender were associated with severe events, defined as those requiring being admitted to the ICU, invasive ventilation, or associated death [12]. This was not observed in our study, and different reasons may explain it: (1) we considered the COVID-19 severity criteria as defined by the WHO; (2) males and females were equally represented in our cohort; and (3) in our study patients were ~10 years older than the patients included in the meta-analysis (72 years vs. 63 years).

Despite cancer, other comorbidities are described as associated with the development of severe COVID-19 disease. In accordance to the WHO, nine risk factors contribute equally to severe disease and higher mortality due to COVID-19: age more than 60 years (increasing with age), diabetes, hypertension, cardiac disease, chronic lung disease, cerebrovascular disease, chronic kidney disease, immunosuppression, cancer, and smoking [21]. Therefore, we evaluated the impact of each variable alone and combined it in COVID-19 cancer patients’ outcomes. Our results demonstrated that 15.7% had no other risk factor than cancer, 84.2% had more than one risk factor, and the maximum of risk factors was six observed in one patient. The univariate analysis has revealed a 3.5-fold increased risk for severe disease and mortality due to COVID-19, in both patients having ≥ 3 risk factors or ≥ 4 risk factors. However, multivariate analysis adjusted to potential confounders, revealed that cancer patients with chronic kidney disease and immunosuppression presented over 20- and 10-folds increased risk to develop severe COVID-19 disease, respectively; cancer patients with cardiac disease and immunosuppression presented over 8- and 5-fold increased risk of mortality due to COVID-19, respectively. Therefore, our results revealed that, in cancer patients, different but concomitant comorbidities appear to be an important factor in severe SARS-CoV-2 infection and COVID-19 mortality. Despite few literature reports about the impact of different and concomitant comorbidities in cancer patients, univariate analysis by H. Zhang et al. revealed that the presence of hypertension and diabetes were associated with increased risk of severe events and no association was observed concerning cardiovascular diseases [12].

In our study patients presented a median ECOG PS of 1, and unfit patients presented a 6-fold increased risk to severe disease and a 4-fold increased risk to mortality due to COVID-19. The literature lacks reports on patients ECOG PS of cancer patients and the risk of COVID-19 disease, making it difficult to compare and interpret our obtained results. In fact, different studies tend to put more emphasis on age rather than the patient’s functional capacity. Moreover, our patients presented a median time of oncological disease of 4 years, solid tumors were the most frequent, and more than half of patients presented early-stage tumors and, therefore, most were under curative treatment. In our cohort, type of cancer, time of oncological disease, stage of cancer, and/or anticancer treatment before SARS-CoV-2 infection were not associated with COVID-19 severity nor to COVID-19 mortality, as previously described [28]. Nevertheless, some literature reports that the severity of SARS-CoV-2 infection in patients is significantly affected by the types of oncological disease, particularly liquid tumors and lung cancer, that have been associated with worse COVID-19 outcomes [16,28]. This may be explained because patients with hematologic cancer have a more compromised immune system than patients with solid tumors and, therefore, these patients have a higher probability of rapidly deteriorating clinical course once infected with SARS-CoV-2 [29]. Malignant or dysfunctional plasma cells, lymphocytes, or white blood cells in hematologic malignancies have decreased immunologic function, which could be the main reason why patients with hematologic cancer have very high severity and death rates [30]. Indeed, all patients with hematologic cancer are prone to the complications of serious infection [13,14,15], which can exacerbate the condition which could have worsened in patients with COVID-19. Particularly in solid tumors, lung cancer is described as associated with a worse COVID-19 disease outcome [28,31] due to decreased lung function, and severe infection may contribute to the worse outcome in this subpopulation [32]. In contrast, there is also literature demonstrating no statistically significant difference in the risk of severe events comparing patients with lung cancer vs. other solid cancer or hematological cancer [12]. In our study, hematologic cancers or cancer at respiratory and intrathoracic organs represent 8.7% and 10.2%, respectively, which may explain the fact that we did not find any association. Nevertheless, an increased risk of severe COVID-19 disease and mortality due to COVID-19 was statistically significantly associated with immunosuppression, independently of age and/or gender. Additionally, the effect of recent cancer treatment, including cytotoxic chemotherapy, on the COVID-19 course is unclear. Two large observational studies found no evidence of increased mortality with recent cytotoxic chemotherapy administration [33,34], in accordance with our results. By contrast, a 205-patient study found an increased risk of death in patients with COVID-19 who received active chemotherapy [31], and a 107-patient study from China found that rates of severe disease were associated with recent chemotherapy [35]. These studies vary in their endpoints and in their statistical methods, which makes comparisons challenging.

As the COVID-19 pandemic overwhelmed healthcare systems worldwide, non-evidenced based decisions had to be made about the treatment of patients with non-COVID-19 diseases such as cancer. Therefore, it is essential to combine data from several international registries and to ensure the collection of new and more comprehensive data during this ongoing pandemic. In particular, more data concerning cancer treatment should be collected. Although COVID-19 patients with cancer may share some epidemiologic features with the general population with this disease, they may also have additional clinical characteristics. The consequences for oncological care are extensive, as the effects of malignancy or cancer treatments on the outcome of COVID-19 are yet unclear. We are aware that the sample size of the population included in our study may limit how representative the results can be if transposed for the whole population. Nevertheless, it provides highlights for future large-scale national comparisons, especially in combining clinical patients’ characteristics with different types of cancer, as well as with particular anticancer treatments.

## 5. Conclusions

Our results demonstrated that unfit and immunosuppressed cancer patients have an increased risk for both severe disease and mortality due to COVID-19; cancer patients with chronic kidney disease have an increased risk for severe COVID-19 disease, and cancer patients with cardiac disease have an increased risk for mortality due to COVID-19. In our study, type of cancer, time of oncological disease, stage of cancer, and/or anticancer treatment were not associated with COVID-19 severity nor to mortality due to COVID-19. Our midsize cohort provides much-needed information on risk factors for severe COVID-19 disease and associated mortality in a Portuguese cancer population. Despite all study limitations appointed, we believe our findings can serve as an informative starting point for further investigation when a larger cohort from a wide range of healthcare providers becomes available. We hope that our findings will help to better protect patients with cancer affected by the ongoing COVID-19 pandemic.

## Figures and Tables

**Table 1:** Clinicopathological characteristics of cancer patients with SARS-CoV-2 infection.

Patients Characteristics	*n* (%)
Gender, *n* = 127	
Male	59 (46.5)
Female	68 (53.5)
Cancer type, *n* = 127	
Solid tumors	116 (91.3)
Digestive organs	35 (27.6)
Breast	31 (24.4)
Urinary tract	22 (17.3)
Respiratory and intrathoracic organs	13 (10.2)
Skin	8 (6.3)
Central nervous system	4 (3.1)
Genital organs	3 (2.4)
Liquid tumors	11 (8.7)
Lymphoma	2 (1.6)
Leukemia	2 (1.6)
Other	7 (5.5)
Tumor stage (solid tumors only), *n* = 115 *	
Primary tumor localized	66 (57.4)
Primary tumor locally advanced	22 (19.1)
Metastatic	27 (23.5)
Cancer treatment, *n* = 120 *	
Adjuvant chemotherapy	32 (26.7)
Palliative chemotherapy	31 (25.8)
Best supportive care	7 (5.8)
No oncological treatment	50 (41.7)
Other comorbidities despite cancer, *n* = 127	
Yes	110 (86.6)
No	17 (13.4)
Groups of patients most at risk, *n* = 127	
Particular patient group	13 (10.3)
Elderly care resident	11 (8.7)
Health professional	2 (1.6)
No particular patient group	114 (89.8)

* Data referring to the available information.

**Table 2:** Clinicopathological characteristics of COVID-19 disease.

Variable	*n* (%)
Risk factors for severe disease	
Age > 60 years	97 (76.4)
Hypertension	82 (64.6)
Diabetes	38 (29.9)
Chronic lung disease	32 (25.2)
Cardiac disease	22 (17.3)
Immunosuppression	18 (14.2)
Cerebrovascular disease with/without Dementia syndrome	10 (7.9)
Chronic kidney disease	10 (7.9)
Smoking	4 (3.1)
Number of risk factors for severe disease	
Cancer only	20 (15.7)
+1	29 (22.8)
+2	33 (26.0)
+3	21 (16.5)
+4	17 (13.4)
+5	6 (4.7)
+6	1 (0.8)
Place of detection	
Emergency environment	66 (52.0)
Screening	42 (33.0)
Hospitalization	19 (15.0)
Internal Medicine	11 (8.7)
General Surgery	4 (3.1)
Pneumology	2 (1.6)
Orthopedics	2 (1.6)
Symptoms	
Asymptomatic	41 (32.3)
Fever	46 (36.2)
Respiratory	62 (48.8)
Cough	50 (39.4)
Dyspnea	30 (23.6)
Digestive	8 (6.3)
Conscience state changes	13 (10.2)
COVID-19 severity category	
Mild	87 (68.5)
Moderate	22 (17.3)
Severe	18 (14.2)
Admitted at Internal Medicine Department	45 (36.2)
Admitted at Intensive Unit Care	2 (1.6)
Mortality	15 (11.8)

**Table 3:** Univariate analysis of clinicopathological characteristics impact in COVID-19 severity and mortality.

Variable	Severe COVID-19 (*n* = 18)			Mortality Due to COVID-19 (*n* = 15)		
	*n* (%)	*p*	OR (95% CI)	*n* (%)	*p*	OR (95% CI)
Gender						
Male (*n* = 59)	8 (13.6)	0.853	1.10 (0.40–3.00)	7 (11.9)	0.986	1.01 (0.34–2.97)
Female (*n* = 68)	10 (14.7)			8 (11.8)		
Other comorbidities despite cancer, *n* (%)						
Yes (*n* = 110)	16 (14.5)	0.760	1.28 (0.26–6.12)	13 (11.8)	0.995	1.00 (0.21–4.90)
No (*n* = 17)	2 (11.8)			2 (11.8)		
ECOG						
≤2 (*n* = 101)	9 (8.9)	**0.002**	**5.41 (1.88–15.6)**	8 (7.9)	**0.011**	**4.28 (1.39–13.23)**
>2 (*n* = 26)	9 (34.6)			7 (26.9)		
Cancer type						
Solid tumors (*n* = 116)	15 (12.9)	0.205	0.40 (0.09–1.66)	14 (12.1)	0.771	1.37 (0.16–11.6)
Liquid tumors (*n* = 11)	3 (27.3)			1 (9.1)		
Tumor stage						
Non-metastatic (*n* = 89)	10 (11.2)	0.571	1.44 (0.41–5.02)	10 (11.2)	0.966	1.03 (0.26–4.06)
Metastatic (*n* = 26)	4 (15.4)			3 (11.5)		
Cancer treatment						
Yes (*n* = 63)	8 (12.7)	0.314	3.15 (0.34–29.3)	7 (11.1)	0.629	0.77 (0.26–2.26)
No (*n* = 57)	9 (15.8)			8 (14.0)		
Risk factors for severe disease, *n* (%)						
Age > 60y (*n* = 97)	17 (17.5)	0.084	6.16 (0.78–48.40)	12 (12.4)	0.726	1.27 (0.34–4.84)
Hypertension (*n* = 82)	12 (14.6)	0.841	1.11 (0.39–3.20)	8 (9.8)	0.337	0.59 (0.20–1.74)
Diabetes (*n* = 38)	7 (18.4)	0.373	1.60 (0.57–4.50)	5 (13.2)	0.759	1.20 (0.38–3.77)
Chronic lung disease (*n* = 32)	4 (12.5)	0.754	0.83 (0.25–2.72)	4 (12.5)	0.889	1.09 (0.32–3.70)
Cardiac disease (*n* = 22)	6 (27.3)	0.061	2.91 (0.95–8.86)	7 (31.8)	**0.003**	**5.66 (1.79–17.89)**
Immunosuppression (*n* = 18)	9 (50.0)	**<0.001**	**11.1 (3.52–35.00)**	6 (33.3)	**0.005**	**5.56 (1.68–18.33)**
Cerebrovascular disease with/without Dementia syndrome (*n* = 10)	1 (10.0)	0.695	0.65 (0.08–5.49)	2 (20.0)	0.411	2.00 (0.38–10.45)
Chronic kidney disease (*n* = 10)	5 (50.0)	**0.003**	**8.00 (2.04–31.4)**	1 (10.0)	0.854	0.82 (0.10–6.95)
Smoking (*n* = 4)	0 (0.00)	----	----	1 (25.0)	0.423	2.60 (0.25–26.7)
Number of risk factors, *n* (%)						
≥3 (*n* = 45)	11 (24.4)	**0.018**	**3.46 (1.24–9.72)**	8 (17.8)	0.130	2.32 (0.78–6.88)
≥4 (*n* = 24)	7 (29.2)	**0.025**	**3.44 (1.17–10.10)**	6 (25.0)	**0.033**	**3.48 (1.10–10.99)**
Patients’ risk-group, *n* (%)						
Elderly care resident and Health professions (*n* = 13)	6 (46.2)	**0.002**	**7.28 (2.10–25.3)**	2 (15.4)	0.675	1.41 (0.28–7.09)
Elderly care residents (*n* = 11)	6 (54.5)	**0.001**	**10.2 (2.70–38.5)**	2 (18.2)	0.513	1.73 (0.34–8.88)
Symptoms						
Fever (*n* = 46)	8 (17.4)	0.435	1.50 (0.54–4.10)	9 (19.6)	**0.049**	**3.04 (1.01–9.18)**
Respiratory (*n* = 62)	14 (22.6)	**0.013**	**4.44 (1.38–14.4)**	9 (14.5)	0.360	1.67 (0.56–5.00)
Cough (*n* = 50)	12 (24.0)	**0.014**	**3.74 (1.30–10.7)**	6 (12.0)	0.958	1.03 (0.34–3.10)
Dyspnea (*n* = 30)	9 (30.0)	**0.007**	**4.19 (1.48–11.85)**	6 (20.0)	0.120	2.44 (0.79–7.55)
Digestive (*n* = 8)	2 (25.0)	0.374	2.15(0.40–11.60)	1 (12.5)	0.950	1.07 (0.12–9.37)
Conscience state changes (*n* = 13)	4 (30.8)	0.082	3.18 (0.86–11.7)	7 (53.8)	**<0.001**	**15.46 (4.19–57.06)**

## Data Availability

Data may be provided upon request.

## Supplementary Materials

The following are available online at https://www.mdpi.com/article/10.3390/ijerph18168552/s1, Table S1: Clinicopathological characteristics of COVID-19 disease.
