# Supplementary material for: The Impact of COVID-19 Pandemic in Portuguese Cancer Patients: A Retrospective Study
Source: Int J Environ Res Public Health. 2021 Aug 13;18(16):8552. doi: 10.3390/ijerph18168552 (PMC8391914; doi:10.3390/ijerph18168552)
Supplement: Supplementary file 1 [file ijerph-18-08552-s001.zip › ijerph-1292441-supplementary.pdf]

Supplementary Table S1 – Clinicopathological characteristics of COVID-19 disease.

Variable	Disease Severity				Mortality	
	Mild n (%)	Moderate n (%)	Severe n (%)		No n (%)	Yes n (%)
**Type of Oncologic Disease**						
Solid tumors (n=116)						
Nervous system, n=1	----	----	1 (100)		----	1 (100)
Head & Neck, n=3	2 (66.7)	1 (33.3)	----		2 (66.7)	1 (33.3)
Respiratory, n=13	11 (84.6)	1 (7.7)	1 (7.7)		13 (100)	----
Breast, n=31	19 (61.3)	5 (16.1)	7 (22.6)		26 (83.9)	5 (16.1)
Gastrointestinal, n=35	28 (80.0)	3 (8.6)	4 (11.4)		32 (91.4)	3 (8.6)
Gynaecologic, n=3	3 (100)	----	----		3 (100)	----
Urologic, n=22	12 (54.5)	8 (36.4)	2 (9.1)		20 (90.9)	2 (9.1)
Skin, n=8	7 (87.5)	1 (12.5)	----		6 (75.0)	2 (25.0)
Liquid tumors (n=11)						
Leukaemia, n=2	1 (50.0)	----	1 (50.0)		2 (100)	----
Lymphoma, n=2	1 (50.0)	1 (50.0)	----		2 (100)	----
Other Haematologic, n=7	3 (42.9)	2 (28.6)	2 (28.6)		6 (85.7)	1 (14.3)
**Disease Stage**						
Localized, n=67	45 (67.2)	15 (22.4)	7 (10.4)		61 (91.0)	6 (9.0)
Locally Advanced, n=22	18 (81.8)	1 (4.5)	3 (13.6)		18 (81.8)	4 (18.2)
Metastatic, n=26	19 (73.1)	3 (11.5)	4 (15.4)		23 (88.5)	3 (11.5)
Not Available, n=12	5 (41.7)	3 (25.0)	4 (33.3)		10 (83.3)	2 (16.7)

n, number; %, percentage.
